# Transcriptional and Catalytic Responsiveness of the Antarctic Fish *Trematomus bernacchii* Antioxidant System toward Multiple Stressors

**DOI:** 10.3390/antiox10030410

**Published:** 2021-03-09

**Authors:** Maria Elisa Giuliani, Alessandro Nardi, Marta Di Carlo, Maura Benedetti, Francesco Regoli

**Affiliations:** Dipartimento di Scienze della Vita e dell’Ambiente, Università Politecnica delle Marche, Via Brecce Bianche, 60131 Ancona, Italy; m.e.giuliani@univpm.it (M.E.G.); a.nardi@staff.univpm.it (A.N.); marta.dicarlo@staff.univpm.it (M.D.C.); f.regoli@univpm.it (F.R.)

**Keywords:** Antarctic fish, trace metals, climate change, multiple stressors, oxidative stress, Nrf2-Keap1

## Abstract

Ocean-warming and acidification jeopardize Antarctic marine species, adapted to cold and constant conditions and naturally exposed to high pro-oxidant pressures and cadmium (Cd) bioavailability. The aim of this study was to investigate if projected temperature increase and pH reduction may affect the accumulation and the effects of Cd in the rockcod *Trematomus bernacchii*. Organisms were exposed for 14 days to six scenarios, combining environmental or increased temperature (−1 °C, +1 °C) and control or reduced pH (8.05, 7.60), either with or without Cd (40 µg/L). Responses in liver and gills were analyzed at different levels, including mRNA and functional measurements of metallothioneins and of a wide battery of antioxidants, integrated with the evaluation of the total antioxidant capacity and onset of oxidative damages. In the gills, metallothioneins and mRNA of antioxidant genes (*nrf2*, *keap1*, *cat*, *gpx1*) increased after Cd exposure, but such effects were softened by warming and acidification. Antioxidants showed slighter variations at the enzymatic level, while Cd caused glutathione increase under warming and acidified scenarios. In the liver, due to higher basal antioxidant protection, limited effects were observed. Genotoxic damage increased under the combined stressors scenario. Overall results highlighted the modulation of the oxidative stress response to Cd by multiple stressors, suggesting the vulnerability of *T. bernacchii* under predicted ocean change scenarios.

## 1. Introduction

The massive anthropogenic emissions of greenhouse gases are affecting global ocean characteristics: the Southern Ocean has a major role in absorbing excess heat and CO_2_ released in the atmosphere [1,2]. The current trend is expected to increase the temperature in the upper Southern Ocean by 1–3 °C by the end of the century [2], and to further exacerbate the challenge of ocean acidification. Polar marine organisms have several adaptations to extreme, cold and stable environmental conditions, thus being considered highly vulnerable to ocean warming and acidification processes. Studies on Antarctic calcifying organisms depicted several biological effects of increased temperature and *p*CO_2_, which include altered development, morphological changes, shell dissolution, reproductive alterations and oxidative unbalance [3,4,5,6,7]. Single and interactive effects of temperature and pH/*p*CO_2_ stress have also been addressed in polar fishes, with developmental delays, altered acclimation capacity, metabolic shifts and oxidative unbalance [8,9,10,11,12,13,14]. In addition to direct effects, the chemical speciation and bioavailability of pollutants, as well as the responsiveness of cellular mechanisms involved in detoxification and organisms’ health maintenance, can be influenced by ocean warming and acidification [15,16,17,18,19,20,21,22,23]. The emerald rockcod *Trematomus bernacchii* is widely used as a bioindicator organism of Antarctic ecosystems due to its ecological relevance, physiological adaptations and ability to accumulate pollutants. This species is also subjected to natural enrichment of cadmium (Cd) concentrations due to the upwelling phenomena in Terra Nova Bay [24,25], and the increased bioavailability of this metal determines tissue Cd burdens typically 10–20-fold higher compared to those of temperate species [26,27]. Cd is known to interfere with the intracellular redox state through indirect mechanisms, i.e., depletion of antioxidant defenses, inhibition of the mitochondrial electron transport chain and alteration of the homeostasis of redox-active metals [28]. Numerous studies have shown that Cd modulates fundamental metabolic pathways in *T. bernacchii*, including xenobiotics biotransformation mechanisms and vitellogenin expression in males [27,29,30,31]. This species is characterized by a sophisticated antioxidant network, as adaptation to the elevated pro-oxidant pressure, typical of the polar environment [26]. One of the main intracellular regulators of antioxidants is the Nrf2-Keap1 system, detected even in Antarctic fish [32]: the transcription factor Nrf2 in basal conditions is inhibited by its partner Keap1, and is activated in the presence of pro-oxidant molecules, leading to the transcription of a number of antioxidant genes [33]. The complexity of the antioxidant network is determined not only by the variety of defense components and modulatory stimuli, but also by the multiple mechanisms of regulation at different cellular levels, from transcriptional to protein and catalytic activity. The mechanisms regulating effects at molecular and cellular levels are not always synchronous and explicit, and differences between gene expression and functional responses have been previously reported in temperate [18,34,35] and polar species [36].

Considering the limited knowledge on the susceptibility of Antarctic organisms to climate changes, the aim of this study was to investigate if warming and pH reduction may affect the sensitivity of *T. bernacchii* to Cd exposure, exploring the potential modulation of antioxidant defenses and cellular responses. Analysis of Cd accumulation in both liver and gills was integrated with a wide panel of biological responses, including mRNA levels of metallothioneins, *nrf2*, *keap1* and other antioxidant genes integrated with functional responses in terms of metallothioneins protein concentration, catalytic activities of individual antioxidants, total antioxidant scavenging capacity, onset of lipid peroxidation processes and genotoxic damages. Overall, this study was expected to increase our knowledge on reciprocal interactions and synergistic effects occurring between multiple stressors at different levels of biological organization in the Antarctic rockcod *T. bernacchii*.

## 2. Materials and Methods

### 2.1. Experimental Design

Antarctic fish, *T. bernacchii*, were sampled in November during the XXIX Italian Antarctic Expedition (2013–2014) from Terra Nova Bay (Ross Sea) and allowed to acclimate for 10 days to laboratory conditions, with running, unfiltered natural seawater at environmental temperature and pH, −1 °C and 8.05, respectively. Organisms were randomly distributed in six tanks (each containing 10 organisms in 80 L), each assigned to one of the following experimental conditions: *CTRL*, control at environmental temperature (−1 °C) and pH (8.05), *Cd*, exposure to 40 μg/L of Cd, −1 °C, pH 8.05, *Ac*, acidification, pH 7.60 and −1 °C, *W*, warm exposure, +1 °C and pH 8.05, *W + Ac*, warm and acidification, +1 °C and pH 7.60, and *W + Ac + Cd*, warm, acidification and Cd exposure, +1 °C, pH 7.60 and 40 μg/L of Cd. Target acidified pH (7.60) was achieved by mixing natural seawater with small aliquots of CO_2_-saturated seawater [21]. Every two days, exposure water was renewed to maintain target pH, temperature and Cd concentration. After 14 days, organisms were anesthetized on ice and thus sacrificed, and gills and livers were rapidly excised, frozen in liquid nitrogen and stored at –80 °C until analyses. Aliquots of gills and blood were maintained in Carnoy’s solution (3:1, methanol:acetic acid) for micronuclei frequency analyses.

### 2.2. Chemical Analyses

Cadmium concentration was evaluated through previously detailed methods [27]. After drying at 70 °C, gills and liver of exposed organisms were digested under pressure with nitric acid in a microwave digestor system (CEM, Mars Systems). Quality assurance and control were assessed on blank samples and reference standard material (Mussel Tissue Standard Reference Material SRM 2977, National Institute of Standards and Technology), which were always within the 95% confidence interval of certified values. Atomic absorption spectrophotometry with electrothermal atomization (Varian AA240Z GTA 120) was used to determine Cd concentration in target tissues. Data are expressed as μg/g dry weight (d.w.).

### 2.3. Transcriptional Analyses

Total RNA was purified from liver and gills after tissues’ homogenization in Trizol^®^ reagent (Sigma-Aldrich), according to the manufacturer’s instructions. The concentration of total RNA was determined using the Nano-Drop ND-1000 UV-Visible Spectrophotometer and the RNA integrity was assessed on an agarose-formaldehyde gel. Total cDNA was synthesized from 1 μg of each RNA sample, using a combination of oligo(dT) and random hexamer primers (iScript cDNA Synthesis Kit, Bio-Rad). The mRNA levels of the following genes were quantified: metallothioneins (*mt*), catalase (*cat*), glutathione peroxidase 1 (*gpx1*), Cu/Zn-superoxide dismutase (*Cu/Zn-sod*), nuclear factor erythroid 2-related factor 2 (*nrf2*) and Kelch-like ECH (Erythroid-derived CNC-Homology factor)-associated protein 1 (*keap1*). Absolute quantitative real-time Polymerase Chain Reactions (qPCR) were performed with specific primer pairs (Table 1) through the SYBR green method in the StepOnePlus^®^ Real-Time PCR System (Applied Biosystems). qPCR reactions were set up in a final 15 μL volume with 7.5 μL of SYBR Select Master Mix (Life Technologies), 5 μL of total cDNA (1:5 in RNAse-free water) and 200 nM of both forward and reverse primer (Table 1). The qPCRs were run with the following method: 2 min at 95 °C (1 cycle), 15 s at 95 °C, 15 s at the annealing temperature (Table 1) and 1 min at 72 °C (40 cycles). A melting analysis was performed at the end of the qPCR method (1 min at 95 °C, 10 s at the annealing T and fluorescence detection at increasing temperatures up to 95 °C) to verify the specificity of amplification.

A non-template control was included to check for the absence of contaminations. For each gene, serial dilutions of known copy numbers of a pGEM plasmid (Promega) containing the gene of interest were used as standards. cDNA samples and standards were amplified in duplicate in the same run. A calibration curve was built with cycle threshold (Ct) values of standards versus corresponding log_10_ copy numbers. Ct values of cDNA samples were converted into copy numbers by interpolating the calibration curve.

### 2.4. Biological Responses

Validated protocols, detailed in Supplementary Material 1 (SM1), were applied for the analysis of the following biomarkers in both gills and livers: concentration of metallothioneins, activities of catalase, glutathione S-transferase, glutathione reductase, total glutathione concentration, total oxyradical scavenging capacity (TOSC) toward peroxyl and hydroxyl radicals and peroxynitrite (ROO^●^, HO^●^, ONOO^−^, respectively) and malondialdehyde levels. Glutathione peroxidases (both Se-dependent and total forms) were analyzed in both gills and livers, but in livers the high variability of obtained results, including those in the control group, caused unreliable responses and results were considered not suitable for publication. Genotoxic parameters (DNA fragmentation and micronuclei frequency) were analyzed in gills and blood.

### 2.5. Statistical Analyses

The null hypothesis that no significant difference existed between experimental conditions for each of the investigated parameters was tested through one-way analysis of variance (ANOVA), followed by Tukey HSD post-hoc tests to compare a group of means when the null hypothesis was rejected (*p* < 0.05). Relationships among the different experimental conditions and each parameter’s contribution to separation were visualized through principal components analysis (PCA). All statistical analyses were conducted in RStudio (version 1.2.5033).

## 3. Results

### 3.1. Cadmium Concentration, Metallothioneins mRNA and Protein Levels

**Figure 1:**
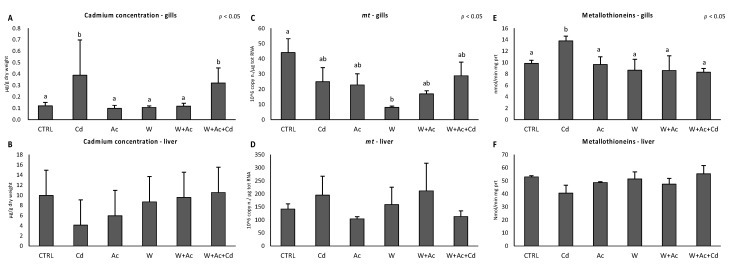
Cadmium concentrations (**A**,**B**, µg/g dry weight), metallothioneins mRNA (**C**,**D**) and protein levels (**E**,**F**) in gills (**A**,**C**,**E**) and livers (**B**,**D**,**F**) of *T. bernacchii* exposed to different experimental conditions. Different letters highlight significant differences between groups of means. Data are expressed as mean ± standard deviations, *n* = 5.

Cd concentration significantly increased in gills of organisms exposed to Cd, dosed alone or in combination with pH and temperature variations, showing values 3- to 5-fold higher than the control group (Figure 1A). On the contrary, no significant variations in Cd levels were observed in liver of fish exposed to Cd, alone and in combination with other factors (Figure 1B). Metallothioneins revealed a significant reduction of mRNA in gills of organisms exposed to temperature increase, while protein levels slightly increased in gills of Cd-exposed fish (Figure 1C,E). Conversely, no significant difference compared to control organisms was measured in livers of exposed organisms, neither for mRNA nor for proteins (Figure 1D,F).

### 3.2. Antioxidants mRNA Levels

**Figure 2:**
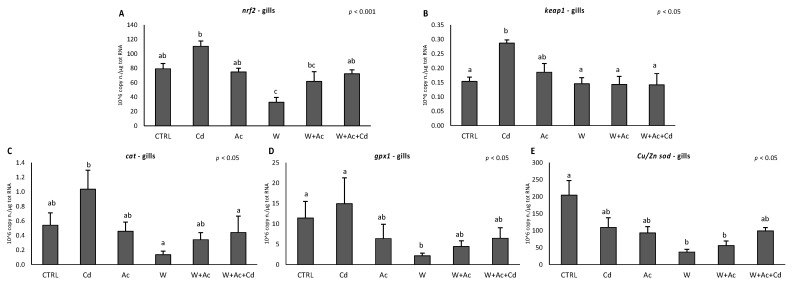
Transcriptional responses of antioxidant regulation system (**A**, *nrf2*; **B**, *keap1*) and antioxidant enzymes (**C**, *cat*; **D**, *gpx1*; **E**, *Cu/Zn-sod*) in gills of *T. bernacchii*. Different letters highlight significant differences between groups of means. Data are expressed as mean ± standard deviations, *n* = 5.

Transcriptional regulation of antioxidant responses was observed in gills. Both *nrf2* and *keap1* were significantly increased by Cd alone, while *nrf2* significantly decreased in the warm condition (Figure 2A,B). Similar trends were observed for *cat* and *gpx1*, with an increase in Cd-exposed organisms and a certain decrease in organisms exposed to higher temperature (Figure 2C,D). *Cu/Zn-sod* decreased in all exposed organisms, with a statistically significant effect in organisms exposed to warm conditions (Figure 2E).

**Figure 3:**
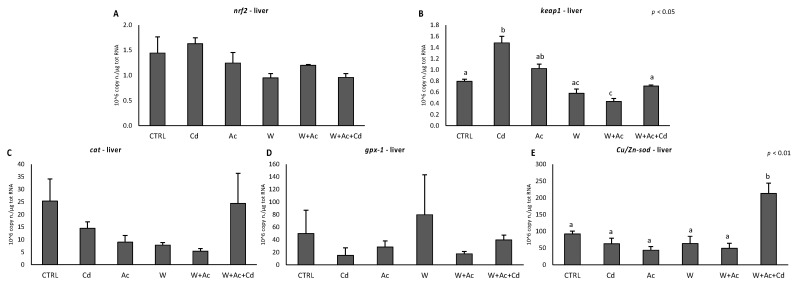
Transcriptional responses of antioxidant regulation system (**A**, *nrf2*; **B**, *keap1*) and antioxidant enzymes (**C**, *cat*; **D**, *gpx1*; **E**, *Cu/Zn-sod*) in liver of *T. bernacchii*. Different letters highlight significant differences between groups of means. Data are expressed as mean ± standard deviations, *n* = 5.

In the liver, no variation occurred for *nrf2*, while *keap1* was enhanced by Cd alone and downregulated by co-exposure to higher temperature and reduced pH (Figure 3A,B). *Cat* showed a decreasing trend in organisms exposed to warming and acidification, alone or in combination (Figure 3C), while more fluctuating variations were observed for *gpx1* after exposure to different conditions (Figure 3D). A marked upregulation of *Cu/Zn-sod* was measured only in organisms co-exposed to Cd in warm and acidified conditions (Figure 3E).

### 3.3. Antioxidant System Functional Analyses

**Figure 4:**
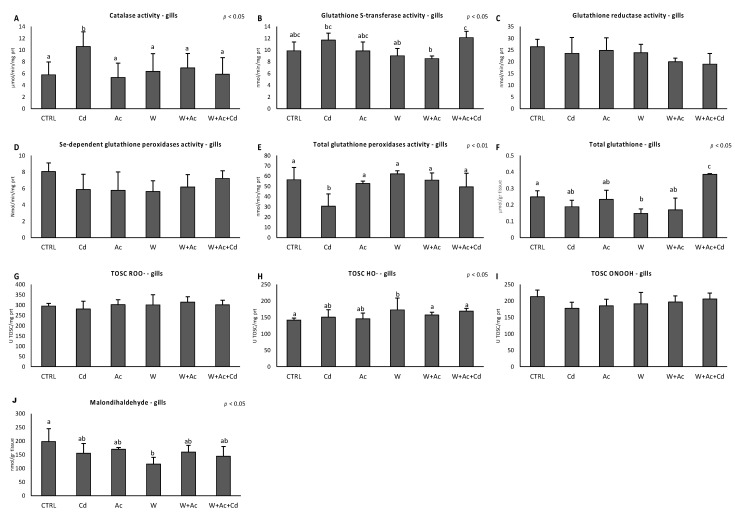
Activities of individual antioxidant enzymes (**A**–**E**) and total glutathione concentration (**F**), Total Ox-yradical Scavenging Capacity (TOSC) toward peroxyl and hydroxyl radicals and peroxynitrite (**G**, TOSC ROO^●^, **H**, TOSC HO^●^ and **I**, TOSC ONOOH, respectively), and malondialdehyde content (**J**) in gills of T. bernacchii exposed to dif-ferent experimental conditions. Different letters highlight significant differences between groups of means. Data are ex-pressed as mean ± standard deviations, *n* = 5.

The antioxidant status was further assessed by functional analyses of various antioxidants integrated with the total capability to neutralize different forms of oxyradicals. Gills exhibited a decrease of total glutathione peroxidases activity after exposure to Cd alone, and total glutathione decreased in organisms exposed to warming and increased in fish co-exposed to all three stressors (Figure 4E,F). In addition, glutathione S-transferase activity under co-occurring warming and acidification was significantly enhanced by Cd (Figure 4B). The variations of individual antioxidants were not paralleled by any significant change in the TOSC values toward ROO^●^, HO^●^ and ONOO^●−^ (Figure 4G–I). Malondialdehyde content was reduced in gills after exposure to warm condition (Figure 4L).

**Figure 5:**
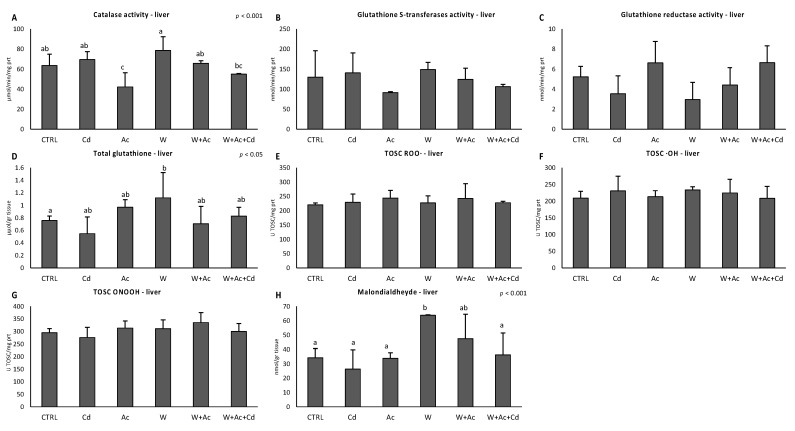
Activities of individual antioxidant enzymes (**A**–**C**) and total glutathione concentration (**D**), Total Oxyradical Scavenging Capacity toward peroxyl and hydroxyl radicals and peroxynitrite (**E**, TOSC ROO^●^, **F**, TOSC HO^●^ and **G**, TOSC ONOOH, respectively), and malondialdehyde content (**H**) in liver of *T. bernacchii* exposed to different experimental conditions. Different letters highlight significant differences between groups of means. Data are expressed as mean ± standard deviations, *n* = 5.

Responses of antioxidants were less evident in liver: catalase showed a slight but significant decrease only in organisms exposed to acidification, alone or in association with Cd and warming (Figure 5A). Temperature increase caused a significant enhancement of total glutathione content (Figure 5D), and no changes occurred for TOSC values (Figure 5E–G). A significant increase of malondialdehyde content was observed in liver of organisms exposed to the warm condition (Figure 5H).

### 3.4. Genotoxic Damage

**Figure 6:**
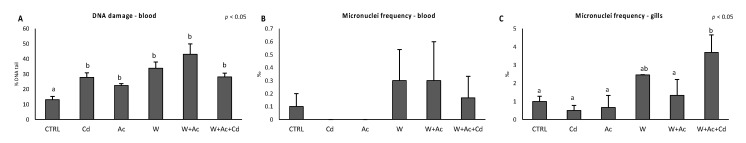
DNA damage and micronuclei frequency, in tissues of *T. bernacchii* exposed to different experimental conditions. Panels (**A**) and (**B**) blood, and (**C**) gills. Different letters highlight significant differences between groups of means. Data are expressed as mean ± standard deviations, *n* = 5.

An increased DNA fragmentation was observed in the blood of all exposed organisms, particularly prominent in organisms exposed to warming conditions (Figure 6A); although not significant, micronuclei frequency also showed a trend of increase in blood of organisms from the warm-treated groups (Figure 6B). A certain role of temperature on micronuclei frequency was evident even in the gills, although significant variations occurred only in organisms co-exposed to all investigated stressors (Figure 6C).

### 3.5. Principal Components Analysis

**Figure 7:**
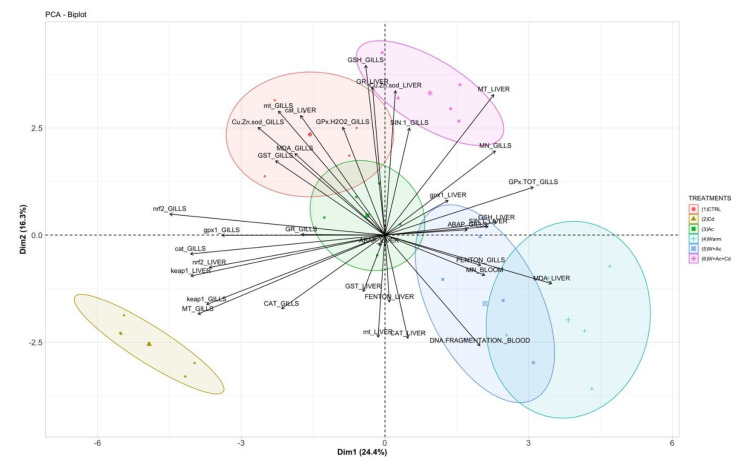
Graphical representation of principal component analysis conducted on the whole dataset of biological parameters.

The principal components analysis on the whole biological parameters’ dataset produced a two-dimensional arrangement (Figure 7), explaining almost 41% of the total variance. A fair partitioning was observed among organisms exposed to Cd alone and all other treatments, and a further discrimination occurred between treatments of warming and acidification with and without Cd. This pattern was mainly determined along the PC1 axis by *nrf2* and *keap1* transcription both in liver and gills, metallothioneins levels and *cat*, *gpx1* and *Cu/Zn-sod* transcription in gills; along the PC2 axis, the pattern was mostly determined by total glutathione in gills, metallothioneins levels and antioxidants in liver.

## 4. Discussion

*T. bernacchii* is a key species of the Antarctic marine environment [26] and understanding its vulnerability to future changes of ocean conditions is fundamental for the preservation of this ecosystem. Naturally exposed to elevated Cd concentrations [37], this species is extremely tolerant to this element, but the impact of a changing environment on such tolerance is still unknown.

Bioaccumulation results highlighted differences between gills and livers: while Cd concentration increased in gills of Cd-exposed organisms (either alone or in combination with other stressors), this was not evident in the liver. This difference could reflect the high basal levels of Cd in liver with values of approximately 10 µg/g in control organisms: these concentrations make a not appreciable variation in Cd content as compared to that observed in the gills. Although temperature and pH variations have been demonstrated to affect metals’ bioavailability for several marine species, our results showed that 14 days of exposure to Cd under thermal and pH/*p*CO_2_ stress did not affect the accumulation of this element in liver and gills of *T. bernacchii*. Similar observations were obtained in polar scallop *Adamussium colbecki*, temperate mussel *Mytilus galloprovincialis* and scallop *Flexopecten glaber* exposed to Cd, and oyster *Crassostrea gigas* exposed to As [4,21,22,38]. These results confirm that effects of increased temperature and acidification on bioavailability and bioaccumulation of pollutants are difficult to generalize, being influenced by intrinsic characteristics of the compound (i.e., chemical speciation) and biological features of the species, such as basal levels, tissue and physiological responsiveness to stressors.

The lack of Cd accumulation in liver was paralleled by similar results on metallothioneins, both in terms of *mt* mRNA and content of these cytosolic proteins. It is worthy to note that their basal levels are extremely high in liver of *T. bernacchii*, supporting their role as adaptation mechanisms to the naturally elevated bioavailability of Cd [26,27]. On the other hand, Cd exposure induced metallothioneins response in gills only at the protein level, suggesting a mismatch between molecular and functional responses, as already observed in *M. galloprovincialis* [18], or a slow turnover rate of proteins typical of cold-adapted organisms [39]. Interestingly, the increase of temperature significantly lowered the basal *mt* transcription in gills, while the co-exposure to Cd, warming and acidification suppressed their response at the protein level despite the consistent Cd accumulation. Such conditions may have accelerated protein recycling, allowing to hypothesize an involvement of metallothioneins, rich in thiolic groups, as antioxidant scavengers [40].

Oxidative stress is amidst the most prominent pathways of metal-induced toxicity [41], and also, future ocean conditions could challenge marine species through oxidative stress: thermal stress has been demonstrated to enhance reactive oxygen species (ROS) production and oxidative damage in several marine species [42,43], and a role of acidification in enhancing oxidative pressure due to mitochondrial alterations has been hypothesized [10,44]. Polar organisms’ vulnerability to oxidative damage is enhanced due to typical adaptive biological traits, such as high polyunsaturated fatty acids content and the consequential susceptibility of biological membranes to peroxidation, relevant mitochondrial density, abundance of lipids stored as energy reserves, slow proteins and RNAs turnover, causing delayed responsiveness to ROS attack [39,45]. In addition, environmental factors can represent sources of oxidative challenge for polar organisms, such as high dissolved oxygen levels in cold seawater, extreme fluctuations of solar radiation, seasonal dynamics of ice melting and primary production. For these reasons, polar species show elevated antioxidant defenses in comparison to their temperate counterparts, either in terms of enzymatic activities or low molecular weight scavengers, as reported for Antarctic scallop *A. colbecki*, polar clam *Laternula elliptica* and *Mya truncata* [46,47,48], and for the fishes *Pagothenia borchgrevinki*, *T. bernacchii* and *Pachycara brachycephalum* [49,50].

In this work, the antioxidant system of *T. bernacchii* was explored at molecular and functional levels through a broad array of biomarkers, including the evaluation of single antioxidant defenses, total antioxidant capacity and onset of oxidative damages. Our results demonstrated higher antioxidant responsiveness to stressors in the gills than in the hepatic tissue. Similar tissue-specific differences were already observed in other species subjected to similar experimental conditions, such as the Antarctic scallop *A. colbecki* and the Mediterranean mussel *M. galloprovincialis* [4,21,23], while the temperate scallop *F. glaber* showed a lower capability to counteract oxidative stress in gills compared to digestive gland [22]. Interestingly, levels of the majority of antioxidants were much higher in the liver than in the gills of *T. bernacchii*; as a consequence, the elevated basal protection toward oxidative insults in the liver may explain the limited responsiveness observed in this tissue. Only the transcription factor *nrf2* was more elevated in the gills, likely to compensate the lower basal antioxidant levels. In this respect, high levels of *nrf2* mRNA would allow a prompt activation of the antioxidant system, ensuring a fast transcription rate in case of pro-oxidant pressure. The role of the Nrf2 system as a protective mechanism towards an increased oxidative challenge was confirmed in this study by the induction of *nrf2* and *keap1* in the gills of Cd-exposed *T. bernacchii*, further supported by the contemporary increase of *cat* and *gpx1* transcripts. Exposure to Cd is known to activate the Nrf2-mediated antioxidant response [51,52], and the coordinated upregulation of *nrf2*, *keap1* and antioxidant genes has already been observed in temperate and Antarctic models [32,53,54]. The increase of the inhibitor *keap1* is considered an autoregulatory feedback loop that controls Nrf2 induction [55]. Although Cd was effectively accumulated in gills, even in co-exposure with warming and acidification (*W* + *Ac* + *Cd*), activation of antioxidant genes did not occur, suggesting that thermal stress and hypercapnia downregulated Cd-induced responsiveness. It is worthy to note that not all antioxidants varied in a synchronous way: *Cu/Zn-sod* was the most sensitive antioxidant transcript in gills, exhibiting a generalized downregulation in all experimental conditions. This antioxidant is responsible for neutralizing superoxide anion, and such a reduction at the transcriptional level, if persistent for prolonged time, may represent a threat for the cells; however, in the gills of organisms co-exposed to Cd, acidification and warming, the presence of other compensation mechanisms in response to Cd could be hypothesized given the scarce variations of antioxidant enzymes, a constant total antioxidant capacity and no indication of oxidative damage. In this respect, the increase of total glutathione could indicate that a certain antioxidant protection is ensured by low molecular weight scavengers compensating the limited responses at the enzymatic level, probably due to different regulatory mechanisms. Gills of organisms exposed to increased temperature also showed lowered levels of several transcripts (*mt*, *nrf2*, *Cu/Zn-sod*, *gpx1*), suggesting a temperature-driven acceleration of mRNA degradation, which is usually slow in species from cold environments [39].

As previously anticipated, the liver of *T. bernacchii* showed limited antioxidants’ responsiveness, with little variations upon exposure to both Cd (in accordance with lack of bioaccumulation) and exposure to increased temperature and *p*CO_2_. The *keap1* mRNA was increased by Cd exposure and decreased in organisms co-exposed to warming and acidification. The lack of *nrf2* induction and the increase of *keap1* could explain the absence of antioxidants’ gene responses in liver of Cd-exposed organisms. Only *Cu/Zn-sod* transcripts markedly increased in organisms exposed to Cd under ocean change scenarios, highlighting synergistic effects of these stressors on gene transcription. At the functional level, however, the number and magnitude of observed variations were limited, probably due to the high basal levels of antioxidants in liver of this species. Such an elevated protection was previously reported as an adaptive strategy of *T. bernacchii* toward the high and fluctuating oxidative pressure experienced by this species during different seasons [26].

A similar lack of induction of antioxidant enzymes was observed in *T. bernacchii*, *T. newnesi* and *P. borchgrevinki* after 7 to 56 days of exposure to higher temperature and acidification [10]; in that study, the authors postulated that basal antioxidants levels in notothens are adequate to compensate the cellular damage induced by tested climate change scenarios. Whether the limited activation of antioxidants in Antarctic fish under global change scenarios should be interpreted as a positive or a negative sign is still to be clarified, since it was shown also in conjunction with a persistent oxidative damage. Antioxidants of *P. borchgrevinki* returned to control levels after a transient response to acute thermal stress, but serious effects occurred on cell functions, disease and ageing [56]. In our study, the onset of genotoxic damage in terms of DNA fragmentation and micronuclei frequency, particularly evident in organisms exposed to all combinations that include warming, confirmed a sustained cellular stress in *T. bernacchii* under tested scenarios; similarly, in temperate organisms, exposure to trace metals and different environmental conditions affected both typologies of genotoxic alterations [13,21,22]. In addition, the higher micronuclei frequency in the gills of co-exposed organisms, coupled with the effects on antioxidants, confirms a certain unbalance of oxyradical metabolism and suggests the vulnerability of *T. bernacchii* to multiple co-occurring stressors. This assumption is further corroborated by principal components analysis, that provides a clear separation between treatments with single and combined stressors and suggests overall divergent biological effects of Cd in *T. bernacchii* at control environmental conditions or under predicted ocean change scenarios.

## 5. Conclusions

In conclusion, new insights on synergistic mechanisms and modulatory effects of ocean warming, acidification and metals exposure were provided in a key Antarctic species. Despite the elevated basal antioxidant protection in *T. bernacchii* as an adaptive mechanism to face the high environmental pro-oxidant pressure, this species is susceptible to interactions between environmental stressors related to climate changes and Cd bioavailability, with specific sensitivities among analyzed tissues toward different factors. Further studies are needed to better understand long-term adaptive mechanisms and joint consequences on physiological status of this species.

## Figures and Tables

**Table 1:** Primer sequences, amplicon sizes, annealing temperatures and accession numbers for each target gene analyzed by qPCR in fish tissues. Fwd: forward primer; Rev: reverse primer; ^a^: size in base pairs. ^b^: temperature in °C.

Gene	Gene Name	Primer Sequences	Amplicon ^a^	Annealing ^b^	Accession Number
*mt*	*metallothioneins*	Fwd: 5′ GTTTGACTTCCTTCATCCCTGTG 3′ Rev: 5′ TGCTGTGTTTGGTTCCTTGG 3′	105	65	AJ011585, Z72485
*cat*	*catalase*	Fwd: 5′ GCCTGATGGTTTCCGTCATA 3′ Rev: 5′ CCTGACATGTTCTTTATGCCTTG 3′	126	62	LT962462
*gpx1*	*glutathione peroxidase 1*	Fwd: 5′ TTGTGTTCCTGAGGGAGATG 3′ Rev: 5′ ACGTCGTTCCTGCATACTG 3′	103	58	XM_034139369
*Cu/Zn-sod*	*Cu/Zn superoxide dismutase*	Fwd: 5′ TTCTTCGAGCAGGAGAATG 3′ Rev: 5′ GATGCACCCGTTTGTATTG 3′	120	56	AY736280
*nrf2*	*nuclear factor erythroid 2-related factor 2*	Fwd: 5′ GAGTGAGAAGAGCGAGAACATC 3′ Rev: 5′ GGAGTATTCGGAGGGAGATAA 3′	127	57	XM_034128735
*keap1*	*Kelch-like ECH associating* *protein 1*	Fwd: 5′ AGCTACCTGGAGGCGTATAA 3′ Rev: 5′ CTTCCTCCAACAGCGTAGAAA 3′	122	62	XM_034129957

## Data Availability

The data presented in this study are available on request from the corresponding author.

## Supplementary Materials

The following are available online at https://www.mdpi.com/2076-3921/10/3/410/s1, Supplementary Material 1.
