# Supplementary material for: Transcriptional and Catalytic Responsiveness of the Antarctic Fish Trematomus bernacchii Antioxidant System toward Multiple Stressors
Source: Antioxidants (Basel). 2021 Mar 9;10(3):410. doi: 10.3390/antiox10030410 (PMC8000868; doi:10.3390/antiox10030410)
Supplement: Supplementary file 1 [file antioxidants-10-00410-s001.pdf]

Supplementary material (SM1)

Standardized protocols described below have been validated in several studies [4; 29; 47].

Metallothioneins were analysed in liver and gills homogenized (1:5) in 20 mM Tris-HCl buffer (pH 8.6), 0.5 M sucrose, 0.006 mM phenylmethylsulfonyl fluoride (PMSF), and 0.01% β-mercaptoethanol and centrifuged at 30000 g for 45 min. After acidic ethanol/chloroform fractionation of tissue supernatants, metallothioneins were quantified by a spectrophotometric assay using reduced glutathione (GSH) as standard.

For measurement of enzymatic antioxidants, tissues (liver and gills) were homogenized (1:5 and 1:3 w:v ratio respectively) in 100 mM K-phosphate buffer (pH 7.5), 0.1 mM phenylmethylsulphonyl fluoride (PMSF), 0.1 mg mL−1 bacitracin, 0.008 TIU mL−1 aprotinin, 1 mg mL−1 leupeptin, 0.5 mg/mL pepstatin, NaCl 2.5%, and centrifuged at 110000 g for 1 h at 4 °C. Measurements were made with a Varian (model Cary 3) spectrophotometer at a constant temperature of 18 °C. Catalase (CAT) was measured by the decrease in absorbance at 240 nm (extinction coefficient, $\varepsilon = 0.04\ mM^{-1}\ cm^{-1}$) due to the consumption of hydrogen peroxide, H_2O_2 (12 mM H_2O_2 in 100 mM K-phosphate buffer pH 7.0). Glutathione reductase (GR) was determined from NADPH oxidation during the reduction of oxidized glutathione, GSSG ($\lambda = 340$ nm, $\varepsilon = 6.22\ mM^{-1}\ cm^{-1}$). The final assay conditions were 100 mM K-phosphate buffer pH 7.0, 1 mM GSSG, and 60 mM NADPH. Glutathione peroxidases (GPx) activities were assayed in a coupled enzyme system where NADPH is consumed by glutathione reductase to convert the formed GSSG to its reduced form (GSH). The decrease of absorbance was monitored at 340 nm (ε = 6.22 mM−1 cm−1) in 100 mM K-phosphate buffer pH 7.5, 1 mM EDTA, 1 mM dithiothreitol, 1 mM sodium azide (NaN3) (for hydrogen peroxide assay), 2 mM GSH, 1 unit glutathione reductase, 0.24 mM NADPH, and 0.5 mM hydrogen peroxide or 0.8 mM cumene hydroperoxide as substrates, respectively, for the selenium-dependent and for the sum of Se-dependent and Se-independent forms. The rate of the blank reaction was subtracted from the total rate. Glutathione S-transferases (GST) were determined at 340 nm using 1-chloro-2,4-dinitrobenzene as substrate (CDNB). The assay was carried out in 100 mM K-phosphate buffer pH 6.5, 1.5 mM CDNB, 1 mM GSH ($\varepsilon = 9.6\ mM^{-1}\ cm^{-1}$). Total glutathione was analysed in samples of liver and gill homogenized (1:5 and 1:3 w:v ratio respectively) in 5% sulfosalicylic acid with 4 mM EDTA, maintained for 45 min on ice and centrifuged at 37,000 g for 15 min. The resulting supernatants were enzymatically assayed.

For measurement of total oxyradical scavenging capacity, tissues (liver and gills) were homogenized (1:5 and 1:3 w:v ratio respectively) in 100 mM K-phosphate buffer (pH 7.5), NaCl 2.5%, and centrifuged at 110000 g for 1 h at 4 °C. The total oxyradical scavenging capacity (TOSC) assay

measures the overall capability of cellular antioxidants to neutralize different forms of artificially generated oxyradicals, thus inhibiting the oxidation of 0.2 mM a-keto-γ-methiolbutyric acid (KMBA) to ethylene gas. Peroxyl radicals (ROO·) were generated by the thermal homolysis of 20 mM 2–2′-azo-bis-(2-methylpropionamidine)-dihydrochloride (ABAP) in 100 mM K-phosphate buffer, pH 7.4. Hydroxyl radicals (·OH) were produced by the Fenton reaction of iron-EDTA (1.8 μM Fe3+, 3.6 μM EDTA) plus ascorbate (180 μM) in 100 mM K-phosphate buffer. Ethylene formation in control and sample reactions was analysed at 10–12 min time intervals by gas-chromatographic analyses and the TOSC values quantified from the equation: TOSC = 100-(∫SA/∫CA × 100), where ∫SA and ∫CA are the integrated areas calculated under the kinetic curves for samples (SA) and control (CA) reactions. For all the samples, a specific TOSC (normalized to content of protein) was calculated by dividing the experimental TOSC values by the relative protein concentration contained in the assay.

The content of malondialdehyde (MDA) was measured in samples homogenized (1:5 and 1:3 w/v respectively for gills and livers) in 20 mM Tris–HCl pH 7.4, centrifuged at 3000 × g for 20 min and then derivatized in 1 ml reaction mixture containing 10.3 mM 1-metyl-2-phenylindole (dissolved in acetonitrile/methanol 3:1), HCl 32%, 100 μl water and an equal volume of sample or standard (standard range 0–6 μM 1,1,3,3-tetramethoxypropane, in 20 mM Tris–HCl, pH 7.4). The tubes were vortexed and incubated at 45 °C for 40 min. Samples were cooled on ice, centrifuged at 15000 × g for 10 min and read spectrophotometrically at 586 nm; levels of MDA were calibrated against a malondialdehyde standard curve and expressed as nmol/g wet weight.

Protein concentrations were measured according to Lowry method, using bovine serum albumin (BSA) as standard. All biochemical biomarkers are expressed as mean values ± standard deviations (n = 5).

The onset of DNA damage was evaluated at molecular level as single strand breaks (SB) by the Comet assay, and at chromosomal level by the frequency of micronuclei. The comet assay was carried out on blood collected from fishes; cells were resuspended in 0.6% low-melting-point agarose and added with a sandwich stratification to glass slides coated with 1% normal-melting-point agarose. After gel solidification, slides were placed into the lysing solution (2.5 M NaCl, 100 mM EDTA, 1% Triton X-100, and 10% DMSO, pH 10) at 4 °C in the dark for 90 min. DNA was unwound in 75 mM NaOH, 10 mM EDTA (pH 13), and the electrophoretic migration was carried out in the same buffer at 1 V/cm for 10 min. Slides were then neutralized for 10 min in 0.4 M Tris, pH 7.5, fixed in cold methanol for 3 min at −20 °C, and dried. After staining with DAPI, 100 randomly selected “nucleoids” per slide, and two replicates per sample, were examined under fluorescence microscopy (200 × magnification; Olympus BX-51), and the captured images (Image-Pro-Plus package) were

analysed through the software Comet Score. The percentage of DNA in the tail was used to estimate the level of DNA fragmentation. For the frequency of micronuclei an aliquot of fish blood and gills rapidly fixed in Carnoy's solution (3:1 methanol: acetic acid), dispersed on glass slides and stained with the fluorescent dye 4′,6-diamidino- 2-phenylindole at 100 ng $mL{-1}$. For each sample 2000 cells with preserved cytoplasm were scored to assess the presence of micronuclei, defined as round structures, smaller than 1/3 of the main nucleus diameter, on the same optical plan and clearly.
